# Determinants of Early Renal Function Recovery After TIPS in Patients With Portal Hypertension

**DOI:** 10.1002/kjm2.70268

**Published:** 2026-07-29

**Authors:** Meng Jia, Yang Luo, Zhao‐Yang Tan, Xiao‐Ling Zhou, Xi‐You Zhang, Jing‐Ying Sun, Ye Du

**Affiliations:** ^1^ Department of Nephrology Beijing Shijitan Hospital, Capital Medical University Beijing China; ^2^ Department of Endocrinology Beijing Shijitan Hospital, Capital Medical University Beijing China

**Keywords:** estimated glomerular filtration rate, portal hypertension, renal function, risk factors, serum creatinine, transjugular intrahepatic portosystemic shunt

## Abstract

Renal dysfunction frequently occurs in patients with portal hypertension and is linked to unfavorable clinical outcomes. Transjugular intrahepatic portosystemic shunt (TIPS) has the potential to enhance renal perfusion through portal decompression; however, factors predicting early improvement in renal function remain insufficiently defined. This retrospective, single‐center study analyzed 188 consecutive patients with portal hypertension who underwent TIPS. Patients receiving renal replacement therapy or with baseline eGFR < 15 mL/min/1.73 m^2^ were excluded. The primary endpoint was early renal function improvement, defined as a > 10% reduction in serum creatinine within 7 days. Early renal function improvement was observed in 27.7% of the cohort. Lower baseline eGFR was independently associated with higher probability of improvement (adjusted OR: 0.980, 95% CI: 0.966–0.993; *p* = 0.004). Neither pre‐ nor post‐TIPS portal venous pressure nor the magnitude of pressure reduction was significantly associated with renal response. Restricted cubic spline analysis indicated a linear inverse relationship between baseline eGFR and improvement probability. The multivariable model demonstrated modest discriminative ability (AUC 0.666). Baseline renal function was associated with early post‐TIPS early renal function improvement, whereas intra‐procedural portal pressure parameters were not predictive. These findings suggest that early renal function improvement following TIPS may predominantly reflect reversible functional impairment rather than established structural kidney injury.

**Abbreviations**
aOR: adjusted odds ratio
AUC: area under the curve
BUN: blood urea nitrogen
CI: confidence interval
DCA: decision curve analysis
eGFR: estimated glomerular filtration rate
ePTFE: expanded polytetrafluoroethylene
IQR: interquartile range
MELD‐Na: Model for End‐Stage Liver Disease incorporating sodium
OR: odds ratio
PVP: portal venous pressure
RAAS: renin‐angiotensin‐aldosterone system
RCS: restricted cubic spline
TIPS: transjugular intrahepatic portosystemic shunt
VIF: variance inflation factor

## 1 Introduction

Portal hypertension represents a frequent sequela of advanced chronic liver disease and is associated with complications including refractory ascites, variceal bleeding, and hepatic hydrothorax [1, 2]. These clinical manifestations are often accompanied by circulatory disturbances and varying degrees of renal dysfunction [1, 3]. Notably, even modest reductions in renal function have been linked to poorer clinical outcomes in this population [4, 5].

Transjugular intrahepatic portosystemic shunt (TIPS) is a well‐established therapeutic intervention for complications arising from portal hypertension [6, 7, 8]. Through reduction of portal pressure, the procedure alters splanchnic hemodynamics and may enhance effective arterial blood volume [7, 8]. Renal function improvement has been reported in a subset of patients following TIPS [9, 10, 11]; however, such responses are not consistent [12, 13]. Despite technically successful portal decompression, early post‐procedural renal changes vary substantially among patients [9, 13].

Renal dysfunction in the context of portal hypertension is multifactorial in origin. In addition to intrinsic kidney disease, functional impairment related to circulatory dysregulation contributes significantly [4]. Consequently, identifying patients who are more likely to experience early renal function improvement after TIPS remains challenging. Understanding baseline clinical characteristics associated with early renal changes may offer practical value in patient evaluation and perioperative management. In particular, a clearer understanding of the relationship between baseline renal function and short‐term renal response may inform clinical decision‐making.

This study aimed to evaluate baseline clinical factors associated with renal function improvement within 7 days following TIPS in patients with portal hypertension. Additionally, the performance of a multivariable model in estimating the probability of early renal function improvement was examined.

## 2 Materials and Methods

### 2.1 Study Design and Participants

This retrospective, single‐center study was conducted at the Interventional Therapy Center of Beijing Shijitan Hospital. Consecutive adult patients who underwent TIPS placement between June 2021 and April 2022 were reviewed. Eligibility criteria included the presence of portal hypertension with established clinical indications for TIPS, along with the availability of complete perioperative laboratory data.

Patients were excluded if they had received renal replacement therapy prior to the procedure, had a baseline estimated glomerular filtration rate (eGFR) below 15 mL/min/1.73 m^2^, or lacked postoperative renal function measurements within the first 7 days. A total of 188 patients met the study criteria and were included in the final analysis.

The study protocol was approved by the Institutional Ethics Committee of Beijing Shijitan Hospital (Ethics approval number: sjtkyll‐lx‐2023(028)). Given the retrospective design and the use of de‐identified data, the requirement for individual informed consent was waived. Written informed consent for the TIPS procedure had been obtained from all patients as part of routine clinical care. The study was conducted in accordance with the ethical principles outlined in the Declaration of Helsinki.

### 2.2 Data Collection

Demographic information, clinical characteristics, and laboratory parameters were obtained from the electronic medical record system. Baseline laboratory values were defined as the most recent measurements recorded within 24 h prior to the TIPS procedure. Postoperative serum creatinine was monitored during the first 7 days, and the lowest value observed within this interval was used to evaluate early renal function improvement. The eGFR was calculated using the Chronic Kidney Disease Epidemiology Collaboration equation [14].

Severity of liver disease was stratified according to the Child–Pugh system and the Model for End‐Stage Liver Disease incorporating sodium (MELD‐Na) score. Baseline urinalysis findings, including proteinuria, hematuria, glycosuria, and urinary casts, were documented when available.

Periprocedural fluid management was not standardized. Instead, it was individualized based on each patient's clinical condition, vital signs, and fluid balance, as determined by the interventional radiology team during daily ward rounds. In patients with refractory ascites, albumin and diuretics were used according to clinical indications. Data on vasoconstrictor use were not available.

### 2.3 TIPS Procedure and Portal Pressure Measurement

All TIPS procedures were carried out using a standardized transjugular technique. TIPS procedures were performed by at least two, and occasionally three, interventional radiologists, with one acting as the primary operator and the other(s) as assistants. Following ultrasound‐guided cannulation of the right internal jugular vein, a 10F sheath was advanced into the right hepatic vein. Access to the portal venous system was then established using a transjugular liver access set (RUPS‐100; Cook Medical, Bloomington, IN, USA). In all cases, an 8‐mm expanded polytetrafluoroethylene (ePTFE)‐covered stent‐graft (VIATORR; W. L. Gore & Associates, Flagstaff, AZ, USA) was deployed. No predefined target for post‐procedural portal pressure reduction was set.

The contrast agents used during the procedure were low‐osmolar nonionic contrast media, including iohexol, ioversol, and iopamidol. The volume of contrast media was not standardized across patients; the median volume was 100 mL. Due to the retrospective nature of this study, complete portal pressure gradient (PPG) data before and after TIPS were not available for statistical analysis; only portal venous pressure (PVP) values were documented.

PVP measurements were obtained using a 5F pigtail catheter positioned within the main portal vein. The electromechanical pressure transducer was calibrated to zero against atmospheric pressure at the level of the right atrium. Measurements were recorded at end‐expiration during brief apnea to minimize respiratory variability. Pressure waveforms were collected for a minimum of 10 s to allow stabilization and to reduce the influence of Valsalva‐related fluctuations. PVP values were documented immediately before (PVP‐pre) and after (PVP‐post) stent placement.

### 2.4 Study Endpoint

The primary endpoint was early improvement in renal function within 7 days following TIPS. This outcome was defined as a relative reduction in serum creatinine exceeding 10% compared to baseline value. The 10% threshold was selected to capture early changes. Patients who did not achieve this level of reduction, required postoperative renal replacement therapy, or died within the seven‐day follow‐up period were categorized as belonging to the non‐improvement group. Sensitivity analyses were additionally conducted using more stringent criteria, defined as reductions in serum creatinine greater than 15% and 20%. Absolute changes in serum creatinine and eGFR were also analyzed.

### 2.5 Statistical Analysis

Continuous variables are expressed as mean ± standard deviation or as median with interquartile range (IQR), depending on the distribution of the data. Categorical variables are presented as frequencies and percentages. Comparisons between groups were performed using the independent samples *t*‐test or Mann–Whitney *U* test for continuous variables, and the chi‐squared test or Fisher's exact test for categorical variables, as appropriate.

Logistic regression analyses were used to identify variables independently associated with early renal improvement. Variables considered for inclusion in the multivariable model comprised those with a *p*‐value < 0.10 in univariate analysis, as well as those deemed clinically significant. Adjusted odds ratios (aOR) with corresponding 95% confidence intervals (CI) are reported. Restricted cubic spline (RCS) analysis was applied to explore potential nonlinear associations between baseline eGFR and the probability of renal improvement. Model discrimination was quantified using the area under the receiver operating characteristic curve (AUC), and internal validation was performed through bootstrap resampling with 1000 iterations. All statistical analyses were conducted using R software (version 4.4.2), with a two‐sided *p*‐value < 0.05 considered indicative of statistical significance.

## 3 Results

### 3.1 Baseline Patient Characteristics and Hemodynamic Outcomes

A total of 188 patients who underwent TIPS were included in the final analysis. Based on the predefined primary endpoint, a reduction in serum creatinine exceeding 10% within the initial 7 days after the procedure, 52 patients (27.7%) were categorized as exhibiting early renal function improvement, whereas 136 patients (72.3%) were classified as not demonstrating improvement.

Baseline demographic and clinical characteristics are summarized in Table 1. No statistically significant differences were observed between the groups with respect to age, sex, underlying etiology of portal hypertension, or the presence of related complications, such as gastrointestinal bleeding and ascites (all *p* > 0.05). Similarly, the prevalence of major comorbid conditions, including hypertension, diabetes mellitus, and malignancy, along with Child–Pugh class, MELD, and MELD‐Na scores, did not differ significantly between groups (all *p* > 0.05). The etiology of liver disease was as follows: hepatitis B virus (HBV) in 84 patients (65 in the non‐improvement group and 19 in the improvement group), hepatitis C virus (HCV) in 6 patients (4 in the non‐improvement group and 2 in the improvement group). None of the HCV patients had received antiviral treatment before TIPS. Autoimmune liver disease (including autoimmune hepatitis and primary biliary cholangitis) was present in 27 patients (17 in the non‐improvement group and 10 in the improvement group). Other etiologies included alcohol‐related liver disease, nonalcoholic fatty liver disease, drug‐induced liver injury, and cryptogenic cirrhosis. No significant differences in etiology distribution were observed between the two groups (all *p* > 0.05).

**TABLE 1:** Baseline characteristics of the study population.

Variable	Overall (*n* = 188)	Renal function non‐improved (*n* = 136)	Renal function improved (*n* = 52)	*p*
Sex: female (%)	61 (32.4)	41 (30.1)	20 (38.5)	0.360
Age (years)	54.00 [47.00, 63.00]	54.00 [46.00, 63.00]	54.50 [48.00, 64.25]	0.522
Weight (kg)	62.00 [55.45, 72.00]	62.00 [55.00, 70.00]	63.00 [59.00, 75.00]	0.515
Height (m)	1.70 [1.62, 1.73]	1.70 [1.63, 1.73]	1.70 [1.60, 1.73]	0.838
BMI (kg/m^2^)	22.45 [20.19, 25.06]	22.27 [19.71, 24.82]	23.61 [20.36, 25.95]	0.249
Etiology of underlying liver disease				0.534
HBV (%)	84 (44.7)	65 (47.8)	19 (36.5)	
HCV (%)	6 (3.2)	4 (2.9)	2 (3.8)	
Autoimmune liver disease (%)	27 (14.4)	17 (12.5)	10 (19.2)	
Alcohol‐related (%)	16 (8.5)	11 (8.1)	5 (9.6)	
NAFLD (%)	16 (8.5)	12 (8.8)	4 (7.7)	
DILI (%)	6 (3.2)	5 (3.7)	1 (1.9)	
Cryptogenic (%)	14 (7.4)	10 (7.4)	4 (7.7)	
Other specified (%)	19 (10.1)	12 (8.8)	7 (13.5)	
Diabetes mellitus (%)	36 (19.1)	27 (19.9)	9 (17.3)	0.850
Hypertension (%)	15 (8.0)	10 (7.4)	5 (9.6)	0.833
Hepatic malignancy (%)	37 (19.7)	28 (20.6)	9 (17.3)	0.763
Infection (%)	101 (53.7)	70 (51.5)	31 (59.6)	0.402
Primary kidney disease^a^ (%)	13 (6.9)	6 (4.4)	7 (13.5)	0.062
Gastrointestinal bleeding (%)	43 (22.9)	28 (20.6)	15 (28.8)	0.312
Pleural effusion (%)	29 (15.4)	16 (11.8)	13 (25.0)	**0.043**
Ascites^b^ (%)	144 (76.6)	104 (76.5)	40 (76.9)	1.000
Child–Pugh ascites score				0.766
None (%)	44 (23.4)	32 (23.5)	12 (23.1)	—
Mild (%)	79 (42.0)	59 (43.4)	20 (38.5)	—
Moderate to Severe (%)	65 (34.6)	45 (33.1)	20 (38.5)	—
Albumin (g/L)	32.60 [30.10, 35.60]	32.90 [30.17, 35.90]	31.80 [29.80, 34.92]	0.462
Serum creatinine (μmol/L)	62.00 [52.00, 75.00]	60.00 [50.00, 71.25]	69.00 [59.00, 91.50]	**0.001**
BUN (mmol/L)	5.52 [4.30, 7.55]	5.24 [4.02, 6.94]	6.72 [5.00, 8.55]	**0.005**
eGFR (mL/min/1.73 m^2^)	103.00 [91.75, 114.00]	106.00 [95.75, 117.00]	97.50 [76.75, 107.00]	**0.001**
Uric acid (μmol/L)	338.50 [262.50, 433.50]	322.00 [246.00, 405.25]	377.00 [307.50, 504.00]	**0.002**
Potassium (mmol/L)	3.94 [3.69, 4.19]	3.92 [3.67, 4.18]	3.99 [3.70, 4.20]	0.523
Sodium (mmol/L)	140.00 [136.75, 142.00]	140.00 [137.00, 142.00]	139.50 [135.75, 142.00]	0.250
Phosphorus (mmol/L)	1.12 [0.97, 1.27]	1.10 [0.95, 1.26]	1.15 [1.01, 1.33]	0.147
Total CO_2_ (mmol/L)	26.00 [25.00, 28.00]	26.00 [25.00, 28.00]	27.00 [25.00, 28.02]	0.531
Total bilirubin (μmol/L)	27.15 [19.20, 37.62]	27.60 [19.15, 37.20]	26.75 [19.42, 44.20]	0.486
Direct bilirubin (μmol/L)	12.90 [8.50, 18.60]	12.90 [8.15, 17.70]	13.00 [9.47, 19.92]	0.319
Indirect bilirubin (μmol/L)	12.85 [8.97, 19.27]	12.85 [9.67, 19.20]	13.25 [8.62, 19.72]	0.976
Total bile acid (μmol/L)	27.65 [12.40, 50.47]	30.95 [14.47, 48.20]	18.55 [8.50, 52.25]	0.151
INR	1.30 [1.20, 1.48]	1.29 [1.20, 1.47]	1.33 [1.19, 1.51]	0.663
PT (s)	14.30 [13.30, 16.33]	14.20 [13.30, 16.12]	14.65 [13.05, 16.75]	0.648
PT prolongation (s)	2.50 [1.50, 4.53]	2.40 [1.50, 4.32]	2.85 [1.25, 4.95]	0.648
APTT (s)	32.85 [30.48, 35.75]	33.00 [30.50, 36.02]	31.90 [29.78, 34.90]	0.288
Thrombin time (s)	16.70 [15.67, 17.90]	16.75 [15.70, 17.90]	16.60 [15.60, 17.83]	0.675
PTA (%)	67.00 [56.75, 75.00]	69.00 [57.00, 75.00]	63.00 [54.75, 76.00]	0.454
Fibrinogen (g/L)	2.09 [1.65, 2.60]	2.12 [1.64, 2.60]	2.03 [1.72, 2.69]	0.914
D‐dimer (ng/mL)	672.50 [327.00, 1710.25]	651.00 [293.75, 1710.25]	683.00 [351.25, 1605.11]	0.586
Child–Pugh score	8.00 [7.00, 9.00]	8.00 [7.00, 9.00]	8.00 [7.00, 9.00]	0.303
Child–Pugh class				0.483
Class A	30 (16.0)	22 (16.2)	8 (15.4)	—
Class B	128 (68.1)	95 (69.9)	33 (63.5)	—
Class C	30 (16.0)	19 (14.0)	11 (21.2)	—
MELD score	11.56 [9.84, 14.35]	11.48 [9.93, 13.26]	12.21 [9.75, 15.78]	0.228
MELD‐Na score	11.95 [9.98, 14.93]	11.86 [10.09, 14.47]	13.18 [9.92, 17.01]	0.164
Baseline PVP (mmHg)	33.00 [29.00, 37.00]	33.00 [29.00, 37.00]	33.00 [28.00, 38.00]	0.913
Post‐procedural PVP (mmHg)	23.00 [19.00, 26.00]	23.00 [19.00, 25.00]	23.00 [20.00, 26.00]	0.273
Reduction in PVP (mmHg)	11.00 [7.00, 14.00]	11.00 [7.00, 14.00]	10.00 [7.00, 12.50]	0.409
Contrast volume (mL)	100.00 [50.00, 100.00]	100.00 [50.00, 100.00]	100.00 [50.00, 150.00]	0.054
Hypotension during surgery (%)	13 (6.9)	11 (8.1)	2 (3.8)	0.521
Contrast‐enhanced CT scan (%)	172 (91.5)	123 (90.4)	49 (94.2)	0.589
Proteinuria (%)	36 (19.1)	28 (20.6)	8 (15.4)	0.546
Hematuria (%)	36 (19.1)	25 (18.4)	11 (21.2)	0.822
Urinary casts (%)	6 (3.2)	3 (2.2)	3 (5.8)	0.350
Glycosuria (%)	16 (8.5)	13 (9.6)	3 (5.8)	0.563

*Note:* Data are presented as median (IQR), *n* (%), or mean ± SD. Bold values indicate variables with statistically significant differences (*p* <  0.05) between the renal‑function‑improved group and the renal‑function‑non‑improved group.

Abbreviations: APTT, activated partial thromboplastin time; BMI, body mass index; BUN, blood urea nitrogen; CT, computed tomography; eGFR, estimated glomerular filtration rate; INR, international normalized ratio; MELD, model for end‐stage liver disease; PPG, portal pressure gradient; PT, prothrombin time; PTA, prothrombin activity.

^a^ Primary kidney disease was defined as clinically diagnosed primary glomerulonephritis or primary nephrotic syndrome.

^b^ Ascites was graded according to Child‐Pugh criteria: none (absent on physical examination and imaging), mild (detectable only by imaging), moderate (visible distension), and severe (marked distension with respiratory compromise). Moderate and severe ascites were combined for analysis due to the small number of patients in the severe category (*n* = 18).

Regarding baseline renal function, 11 patients (5.9%) had an eGFR below 60 mL/min/1.73 m^2^, including 6 (4.4%) in the non‐improvement group and 5 (9.6%) in the improvement group (*p* = 0.317).

In contrast, the improvement group demonstrated significantly higher baseline levels of serum creatinine, blood urea nitrogen (BUN), and uric acid (*p* < 0.01 for all comparisons). Consistent with these findings, median baseline eGFR was significantly lower in this group compared to the non‐improvement group (97.5 vs. 106.0 mL/min/1.73 m^2^; *p* = 0.001). The median absolute change in serum creatinine was −1.00 μmol/L (IQR: −6.00 to 2.00) in the non‐improvement group and −14.00 μmol/L (IQR: −20.00 to −8.75) in the improvement group (*p* < 0.001). Preexisting pleural effusion was also more frequently observed among patients with renal improvement (25.0% vs. 11.8%; *p* = 0.043). No significant disparities were found in baseline urinalysis findings, including the presence of proteinuria, hematuria, glycosuria, or urinary casts (all *p* > 0.05).

Technical success was achieved in all cases. Across the cohort, median PVP decreased from 33.0 mmHg (IQR: 29.0–37.0) prior to the procedure to 23.0 mmHg (IQR: 19.0–26.0) afterward (*p* < 0.001). However, baseline PVP, post‐procedural PVP, and the extent of pressure reduction were comparable between groups (all *p* > 0.05).

### 3.2 Determinants of Renal Functional Recovery

Univariate analysis identified several variables associated with early renal functional recovery, including baseline eGFR, serum creatinine, BUN, uric acid, MELD‐Na score, and the presence of pleural effusion (all *p* < 0.05). In the subsequent multivariable logistic regression analysis, performed using a stepwise selection process, baseline eGFR remained a stable independent predictor of renal improvement (OR: 0.980; 95% CI: 0.966–0.993; *p* = 0.004).

Although the MELD‐Na score and pleural effusion did not meet conventional thresholds for statistical significance in the primary multivariable model (*p* = 0.144 and *p* = 0.106, respectively), both variables were retained due to their clinical relevance and their contribution to the overall model structure (Table 2).

**TABLE 2:** Univariate and multivariate logistic regression analysis for identifying predictors of improvement.

Variable	Univariate analysis		Multivariate analysis		
	OR (95% CI)	*p*	OR (95% CI)	*p*	VIF
Intercept			1.150 (0.184–7.490)	0.882	—
Age (years)	1.007 (0.983–1.033)	0.553	—	—	—
Sex: Female	1.448 (0.736–2.815)	0.277	—	—	—
eGFR (mL/min/1.73 m^2^)	0.978 (0.964–0.991)	**0.001**	0.980 (0.966–0.993)	**0.004**	1.021
Uric acid (μmol/L)	1.003 (1.001–1.005)	**0.005**	—	—	—
Serum creatinine (μmol/L)	1.015 (1.004–1.028)	**0.009**	—	—	—
BUN (mmol/L)	1.110 (1.022–1.225)	**0.024**	—	—	—
MELD‐Na score	1.096 (1.019–1.182)	**0.014**	1.060 (0.979–1.150)	0.144	1.079
Primary kidney disease	3.370 (1.066–10.980)	**0.037**	—	—	—
Pleural effusion	2.500 (1.092–5.659)	**0.028**	2.050 (0.844–4.900)	0.106	1.057

*Note:* Bold values represent variables with statistical significance (*p* < 0.05) in univariate or multivariate logistic regression analyses.

Abbreviations: BUN, blood urea nitro gen; eGFR, estimated glomerular filtration rate; MELD‐Na, model for end‐stage liver disease‐Na; VIF, variance inflation factor.

Sensitivity analyses applying more stringent definitions of renal improvement, specifically reductions in serum creatinine exceeding 15% and 20%, yielded findings consistent with those of the primary analysis, with baseline eGFR maintaining its association with early renal function improvement. When the most restrictive criterion (> 20% reduction) was applied, the MELD‐Na score reached statistical significance as an independent determinant (Table S1).

Assessment of multicollinearity using the variance inflation factor (VIF) demonstrated values below 2 for all variables, indicating the absence of meaningful collinearity. A correlation matrix illustrating relationships among baseline renal parameters is presented in Figure S1.

### 3.3 Dose–Response Relationship Between Baseline eGFR and Improvement Probability

**FIGURE 1:**
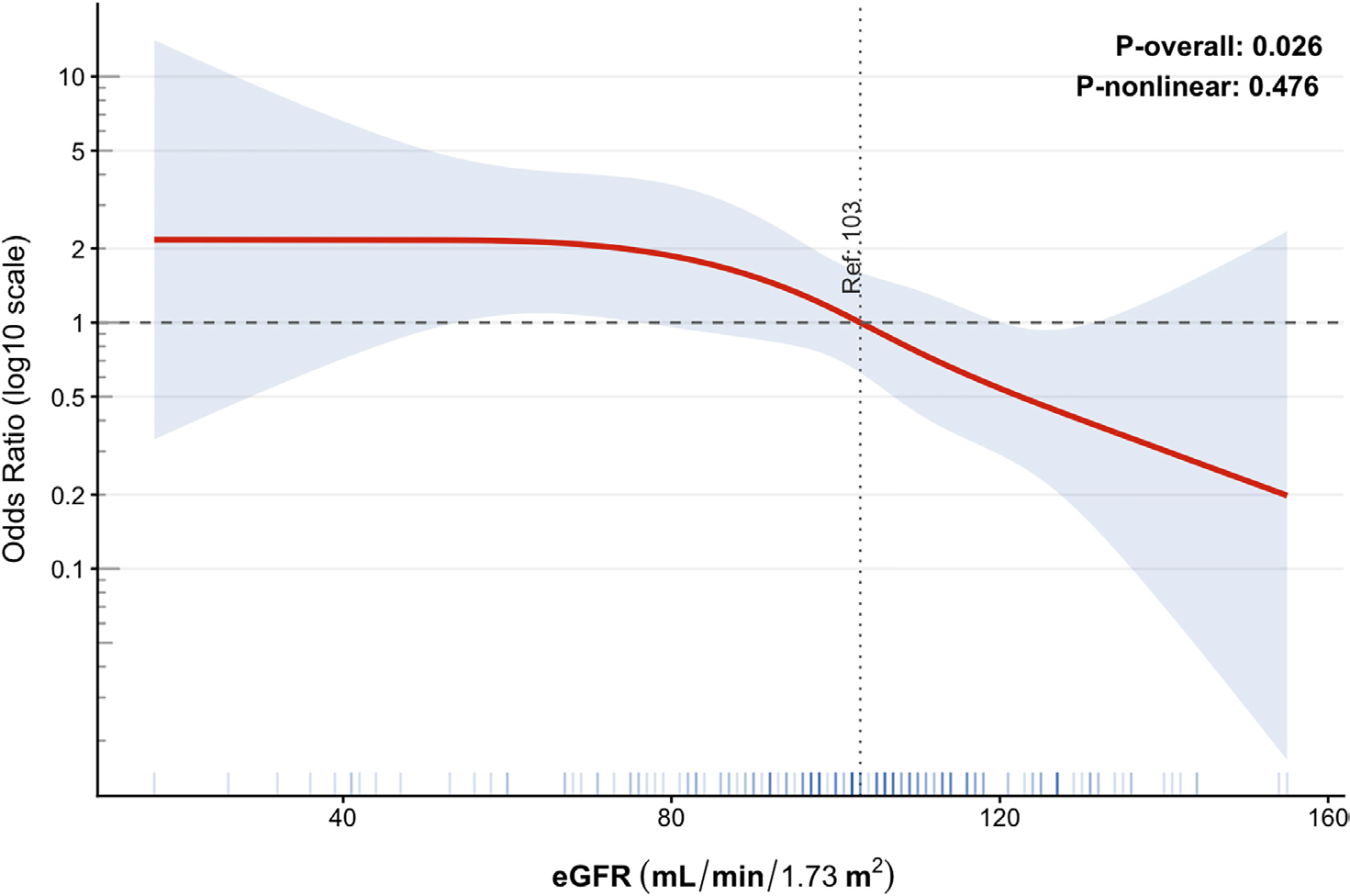
Restricted cubic spline analysis of the association between baseline eGFR and the likelihood of renal function improvement after TIPS. The solid red line represents the multivariate‐adjusted odds ratio (OR) for renal function improvement, and the gray shaded area represents the 95% confidence interval (CI). The model was adjusted for MELD‐Na score and pleural effusion. The vertical dashed line indicates the reference baseline eGFR value (103 mL/min/1.73 m^2^), where the OR is set to 1.0.

RCS analysis was employed to characterize the association between preoperative renal function and the likelihood of early renal function improvement. After adjustment for MELD‐Na score and the presence of pleural effusion, a monotonic linear relationship was observed (*p_overall* = 0.026). There was no indication of nonlinearity across the range of eGFR values (*p_non‐linearity* = 0.476) (Figure 1). Stratified analyses according to baseline eGFR levels are presented in Table S2.

### 3.4 Exploratory Prognostic Nomogram

**FIGURE 2:**
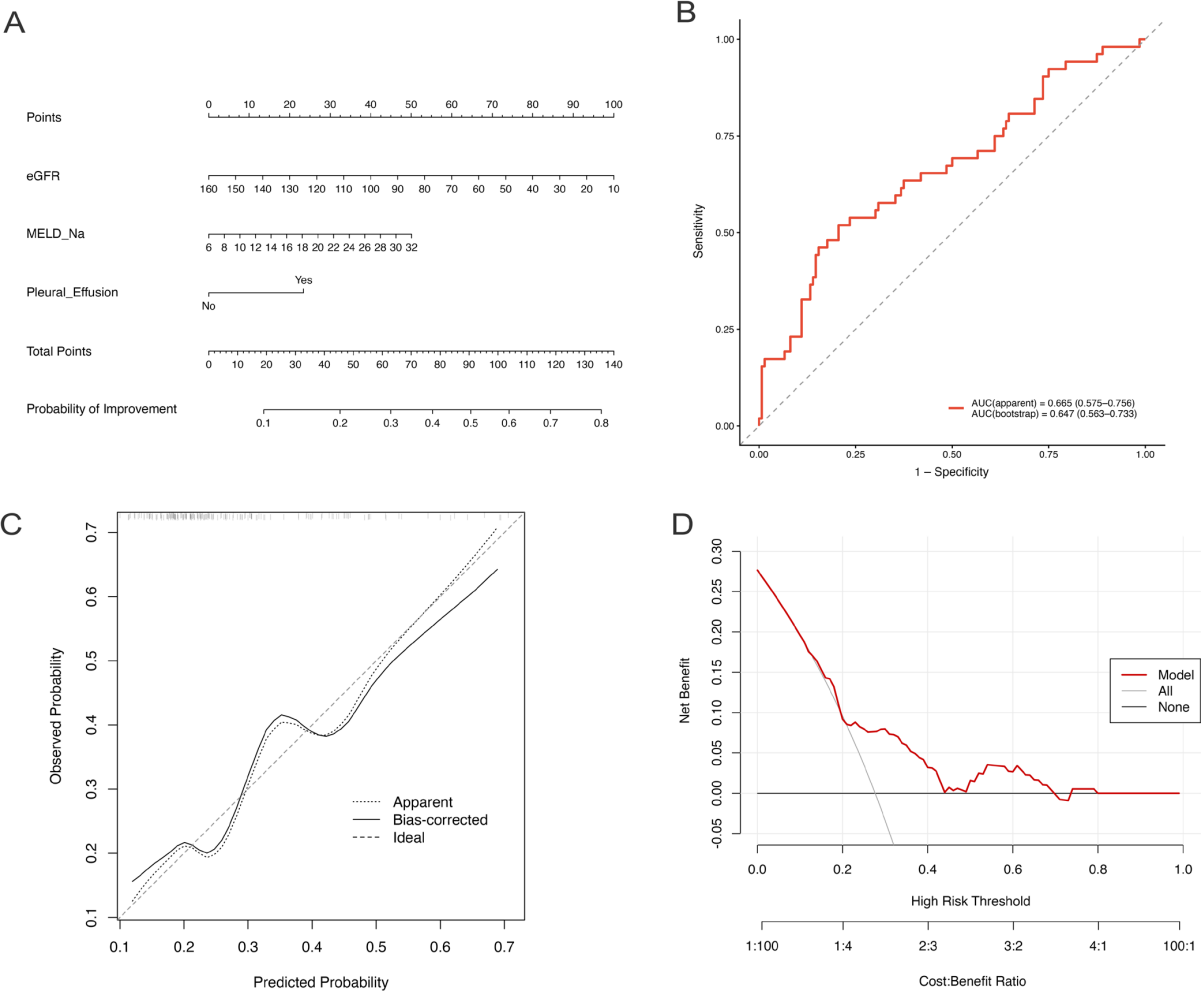
Construction and validation of a nomogram for predicting renal function improvement after TIPS: (A) Nomogram for predicting the probability of renal function improvement. The nomogram was established based on eGFR, MELD‐Na score, and pleural effusion. (B) Receiver operating characteristic (ROC) curve of the nomogram. (C) Calibration curve of the nomogram. The x‐axis represents the predicted probability, and the y‐axis represents the observed probability. The diagonal dashed line represents the ideal prediction. The solid line represents the bias‐corrected performance. (D) Decision curve analysis (DCA) of the nomogram. The red line represents the nomogram model.

To facilitate clinical risk stratification, a preoperative nomogram was developed incorporating baseline eGFR, MELD‐Na score, and the presence of pleural effusion (Figure 2A). The model demonstrated modest discriminative performance, with an AUC of 0.666 (95% CI: 0.576–0.755) (Figure 2B). Following internal validation using bootstrap resampling (1000 iterations), the corrected AUC was 0.647.

Calibration analysis indicated acceptable agreement between predicted probabilities and observed outcomes (Figure 2C). In addition, decision curve analysis (DCA) demonstrated a net clinical benefit across a range of threshold probabilities (Figure 2D). Receiver operating characteristic analyses for models using more stringent definitions of early renal function improvement are presented in Figures S2 and S3.

## 4 Discussion

In this cohort, early improvement in renal function within 7 days following TIPS was documented in approximately 28% of patients. Baseline eGFR demonstrated an inverse association with early renal function improvement. By contrast, no significant differences were observed between groups in baseline PVP, post‐procedural pressure, or the magnitude of pressure reduction. These observations suggest that, although TIPS reliably lowers PVP, the degree of early renal improvement is more closely related to preprocedural renal function.

This study examined early (7‐day) renal function changes after TIPS. We quantified a linear dose–response relationship between baseline eGFR and the probability of early renal function improvement using RCS analysis. We developed a nomogram incorporating baseline eGFR, MELD‐Na, and pleural effusion. We found that intra‐procedural portal pressure parameters were not associated with early renal changes.

Differences in reported renal improvement rates after TIPS across studies may be attributed to several factors. Patient selection varies: some cohorts predominantly include liver transplant candidates [9], whereas others focus on patients with hepatorenal syndrome [11]. Definitions of renal improvement range from complete normalization of serum creatinine to relative reductions of 10%–20%. Timing of assessment ranges from 7 days to 6 months post‐procedure. Lang et al. [9] reported a 25.6% improvement rate at 3 months using a 20% creatinine reduction threshold. Our study observed a 27.7% rate at 7 days using a 10% threshold.

Renal dysfunction in portal hypertension is largely attributed to splanchnic vasodilation and effective arterial underfilling, which in turn promotes activation of the renin–angiotensin–aldosterone system (RAAS) and subsequent renal vasoconstriction [4, 5]. TIPS partially mitigates these pathophysiological changes by redistributing splanchnic blood volume toward the central circulation and attenuating vasoconstrictor activity [11, 15, 16]. Within this framework, patients with lower baseline eGFR may have a greater proportion of functional renal impairment. Following portal decompression, restoration of effective arterial blood volume may facilitate early improvement in glomerular filtration.

In contrast, patients with preserved baseline renal function may exhibit limited capacity for further measurable improvement [9]. The lack of significant differences in baseline urinalysis findings between groups supports the interpretation that functional hemodynamic alterations, rather than overt structural kidney injury, predominantly underlie the observed renal response.

RCS analysis demonstrated a linear inverse relationship between baseline eGFR and the probability of renal improvement across the observed range. There was no indication of nonlinearity across the range of eGFR values. This finding should be interpreted in the context of the study population, in which patients with eGFR values below 15 mL/min/1.73 m^2^ were excluded. Within this selected cohort, renal dysfunction is more likely to represent a continuum of hemodynamic impairment rather than advanced structural kidney damage. The absence of a nonlinear threshold may reflect the limited range of disease severity represented, rather than uniform biological behavior across all stages of renal impairment. Stratified analyses did not demonstrate significant interaction effects across categories of baseline renal function, although the number of events within subgroups was limited.

In this cohort, PVP and its immediate reduction following TIPS were not significantly associated with early renal function improvement. Although portal decompression serves as the primary mechanism for redistributing splanchnic blood volume into the systemic circulation and alleviating neurohumoral vasoconstriction, subsequent improvement in renal perfusion is not determined solely by the magnitude of pressure reduction [17, 18]. The systemic hemodynamic response after TIPS reflects a complex interaction among increased venous return, changes in cardiac output, and recalibration of the renin–angiotensin–aldosterone system [10].

The lack of a detectable association suggests that single‐point intra‐procedural pressure measurements may not adequately capture the dynamic process of postoperative circulatory adaptation [19, 20]. This observation aligns with prior studies indicating that immediate post‐TIPS pressure measurements do not reliably predict portal hemodynamics during follow‐up, partly due to variability in right atrial pressure, which substantially influences pressure gradient fluctuations independent of portal vein pressure reduction [21, 22]. Accordingly, changes in renal function likely arise from multiple interacting factors, in which the effects of portal decompression are modulated by baseline cardiac reserve and the extent of underlying circulatory dysfunction. These findings indicate that intra‐procedural hemodynamic parameters alone have limited utility in predicting short‐term renal outcomes.

The incorporation of the MELD‐Na score and pleural effusion into the multivariable model suggests that markers of systemic decompensation are associated with renal responsiveness. Within the MELD‐Na framework, hyponatremia reflects advanced neurohumoral activation and effective arterial underfilling [23, 24, 25, 26]. In a similar manner, pleural effusion represents a manifestation of significant circulatory dysfunction [27, 28]. As TIPS may mitigate these abnormalities through suppression of neurohumoral activity and modification of systemic hemodynamics, their presence may identify a clinical phenotype characterized by potentially reversible functional renal impairment [9].

The stability of these associations was further evaluated through sensitivity analyses. Baseline eGFR remained a consistent predictor across all thresholds of renal improvement, whereas the MELD‐Na score achieved statistical significance only when a more stringent definition was applied (serum creatinine reduction > 20%). A sensitivity analysis using absolute creatinine change (defined as a decrease of ≥ 0.1 mg/dL) also showed consistent results. This pattern suggests that the influence of systemic disease severity may become more evident in cases demonstrating more pronounced functional recovery. Such findings do not conflict with the established association between hepatic hydrothorax and adverse long‐term outcomes [27, 29]; rather, they underscore the distinction between short‐term hemodynamic reversibility and long‐term prognosis. In addition, these results reflect renal responses under standard perioperative management and do not diminish the importance of established renal protective strategies in clinical care.

An exploratory nomogram was developed to aid clinical stratification, with baseline eGFR contributing the largest weight within the scoring system. The model demonstrated modest discriminative ability (AUC 0.666), consistent with the multifactorial determinants of renal dynamics in cirrhosis. Calibration analysis demonstrated acceptable concordance between predicted and observed probabilities, while DCA indicated a favorable net clinical benefit. This tool is intended to support, rather than replace, clinical judgment by providing a structured method for estimating the likelihood of early renal function improvement following TIPS.

Several limitations should be acknowledged. First, the retrospective design and single‐center setting may restrict the generalizability of the findings. Second, renal function was assessed using serum creatinine‐based eGFR, which can be affected by reduced muscle mass in patients with advanced liver disease [30, 31, 32]. Third, the analysis was confined to short‐term changes within a seven‐day period; the extent to which early renal function improvement correlates with long‐term outcomes remains uncertain. Fourth, only three patients had baseline eGFR below 15 mL/min/1.73 m^2^, which precluded a meaningful sensitivity analysis in this subgroup. Fifth, the exclusion of patients with baseline eGFR < 15 mL/min/1.73 m^2^ limits the generalizability of our findings to those with end‐stage renal disease. Sixth, a mathematical association between baseline eGFR and the outcome definition exists because eGFR is derived from baseline serum creatinine. A percentage reduction may favor patients with lower baseline eGFR. However, this definition was chosen because absolute changes would disproportionately reflect high baseline creatinine levels; absolute change analysis confirmed consistent results. Seventh, data on vasoconstrictor use were not available in this retrospective cohort. Eighth, the modest AUC indicates that renal response following TIPS is likely influenced by parameters beyond baseline physiological factors. Accordingly, these findings should be regarded as hypothesis‐generating and require external validation in independent cohorts.

## 5 Conclusions

Baseline renal function is independently associated with early improvement in renal function within 7 days following TIPS in patients with portal hypertension. Lower preoperative eGFR is linked to a higher probability of early renal function improvement, whereas intra‐procedural portal pressure metrics do not appear to be predictive of early renal changes. These observations suggest that baseline physiological reserve, rather than the immediate magnitude of portal decompression, plays a central role in short‐term renal response. Prospective studies are needed to further define the long‐term clinical significance of these findings and to evaluate the role of preprocedural risk stratification in guiding perioperative management.

## Funding

This work was supported by the Science and Technology Research and Development Program of China State Railway Group Co. Ltd. (Grant No. J2022Z608).

## Ethics Statement

The study was conducted in accordance with the Declaration of Helsinki, and approved by the Institutional Review Board (or Ethics Committee) of Beijing Shijitan Hospital, Capital Medical University (protocol code: sjtkyll‐lx‐2023(028) and date of approval: 29 March 2023). Owing to the retrospective design and use of anonymized data, informed consent for study participation was waived. Written informed consent for the TIPS procedure had been obtained from all patients as part of routine clinical practice.

## Conflicts of Interest

The authors declare no conflicts of interest.

## Supporting information


**Table S1:** Sensitivity analysis of the multivariate logistic regression model for predicting renal function improvement using stricter thresholds.
**Table S2:** Stratified multivariate logistic regression analysis of predictors for renal function improvement based on baseline eGFR levels.
**Figure S1:** Correlation matrix of baseline renal function markers.
**Figure S2:** Validation of the fixed multivariate model (derived from the > 10% improvement threshold) for predicting stricter renal recovery outcomes.
**Figure S3:** ROC curves of the multivariate models refitted for stricter definitions of renal recovery.

## Data Availability

All data generated or analysed during this study are included in this article. Further enquiries can be directed to the corresponding author.
