# Supplementary material for: Determinants of Early Renal Function Recovery After TIPS in Patients With Portal Hypertension
Source: Kaohsiung J Med Sci. 2026 Jul 29:e70268. Online ahead of print. doi: 10.1002/kjm2.70268 (PMC13417660; doi:10.1002/kjm2.70268)

**Supplementary materials**

**Supplementary Table 1.** Sensitivity analysis of the multivariate logistic regression model for predicting renal function improvement using stricter thresholds.

| Variable | Improvement > 15% (N = 39 events) | | | Improvement > 20% (N = 23 events) | | |
| --- | --- | --- | --- | --- | --- | --- |
|  | OR (95% CI) | *P* | VIF | OR (95% CI) | *P* | VIF |
| (Intercept) | 0.938 (0.131–7.067) | 0.95 | - | 0.168 (0.016–1.767) | 0.134 | - |
| eGFR | 0.976 (0.961–0.991) | 0.002 | 1.031 | 0.976 (0.958–0.993) | 0.006 | 1.039 |
| MELD-Na score | 1.075 (0.986–1.169) | 0.094 | 1.107 | 1.150 (1.042–1.271) | 0.005 | 1.093 |
| Pleural effusion | 1.495 (0.546–3.818) | 0.413 | 1.076 | 1.876 (0.561–5.646) | 0.279 | 1.070 |

CI, confidence interval; eGFR, estimated glomerular filtration rate; MELD-Na, model for end-stage liver disease-sodium; OR, odds ratio; VIF, variance inflation factor.

**Supplementary Table 2.** Stratified multivariate logistic regression analysis of predictors for renal function improvement based on baseline eGFR levels.

| Variable | eGFR < 90 mL/min/1.73 m² (N = 43, Events = 19) | | | eGFR ≥ 90 mL/min/1.73 m² (N = 145, Events = 33) | | |
| --- | --- | --- | --- | --- | --- | --- |
|  | OR (95% CI) | P | VIF | OR (95% CI) | P | VIF |
| (Intercept) | 0.081 (0.001–3.926) | 0.226 | - | 5.510 (0.173–247.754) | 0.354 | - |
| eGFR | 0.997 (0.959–1.038) | 0.898 | 1.345 | 0.970 (0.937–1.001) | 0.071 | 1.037 |
| MELD-Na score | 1.172 (1.011–1.409) | 0.053 | 1.246 | 1.021 (0.909–1.138) | 0.706 | 1.109 |
| Pleural effusion | 2.438 (0.385–16.192) | 0.335 | 1.097 | 1.739 (0.560–5.048) | 0.318 | 1.105 |

CI, confidence interval; eGFR, estimated glomerular filtration rate; MELD-Na, model for end-stage liver disease-sodium; OR, odds ratio; VIF, variance inflation factor.

**Supplementary Figure 1.** Correlation matrix of baseline renal function markers.

BUN, blood urea nitrogen; eGFR, estimated glomerular filtration rate; Scr, serum creatinine; UA, uric acid.


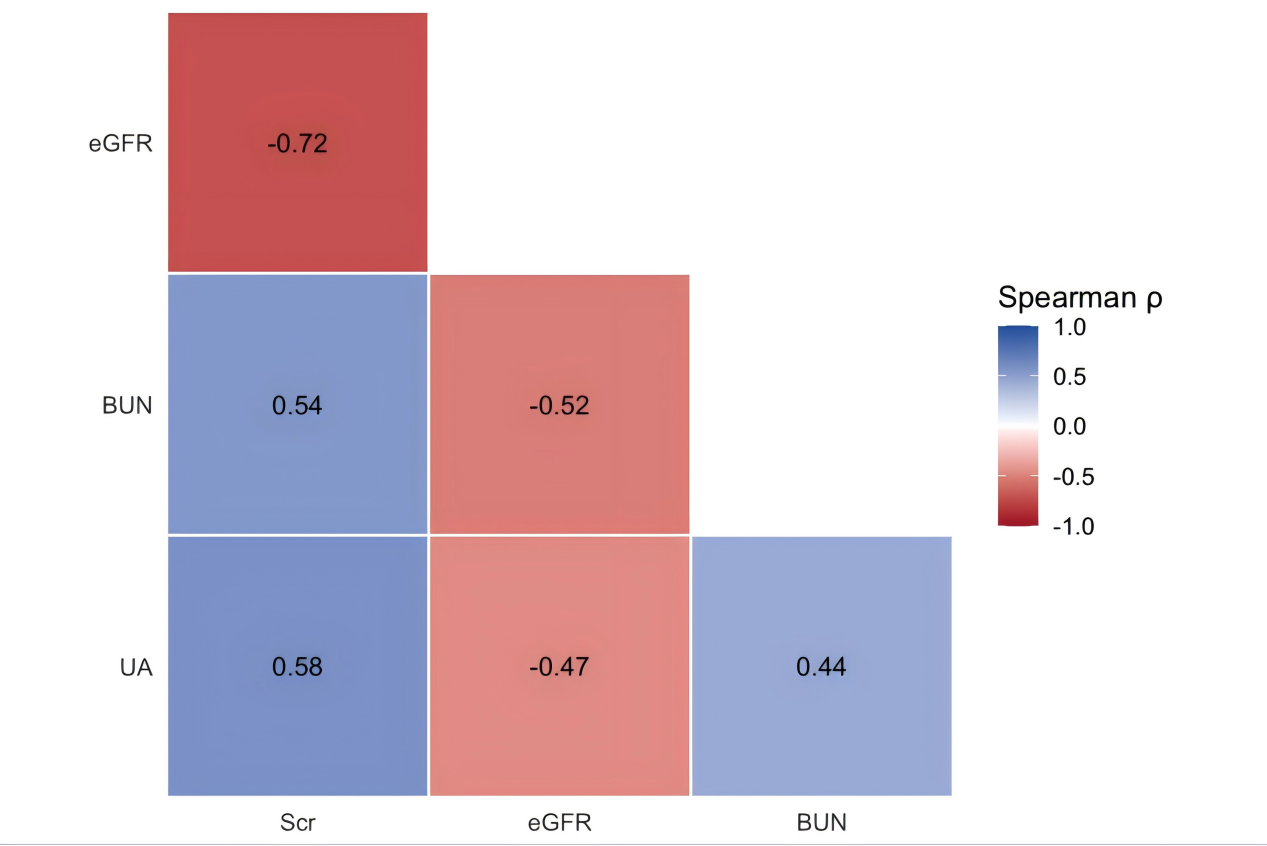


**Supplementary Figure 2.** Validation of the fixed multivariate model (derived from the >10% improvement threshold) for predicting stricter renal recovery outcomes.

AUC, area under the curve; CI, confidence interval.


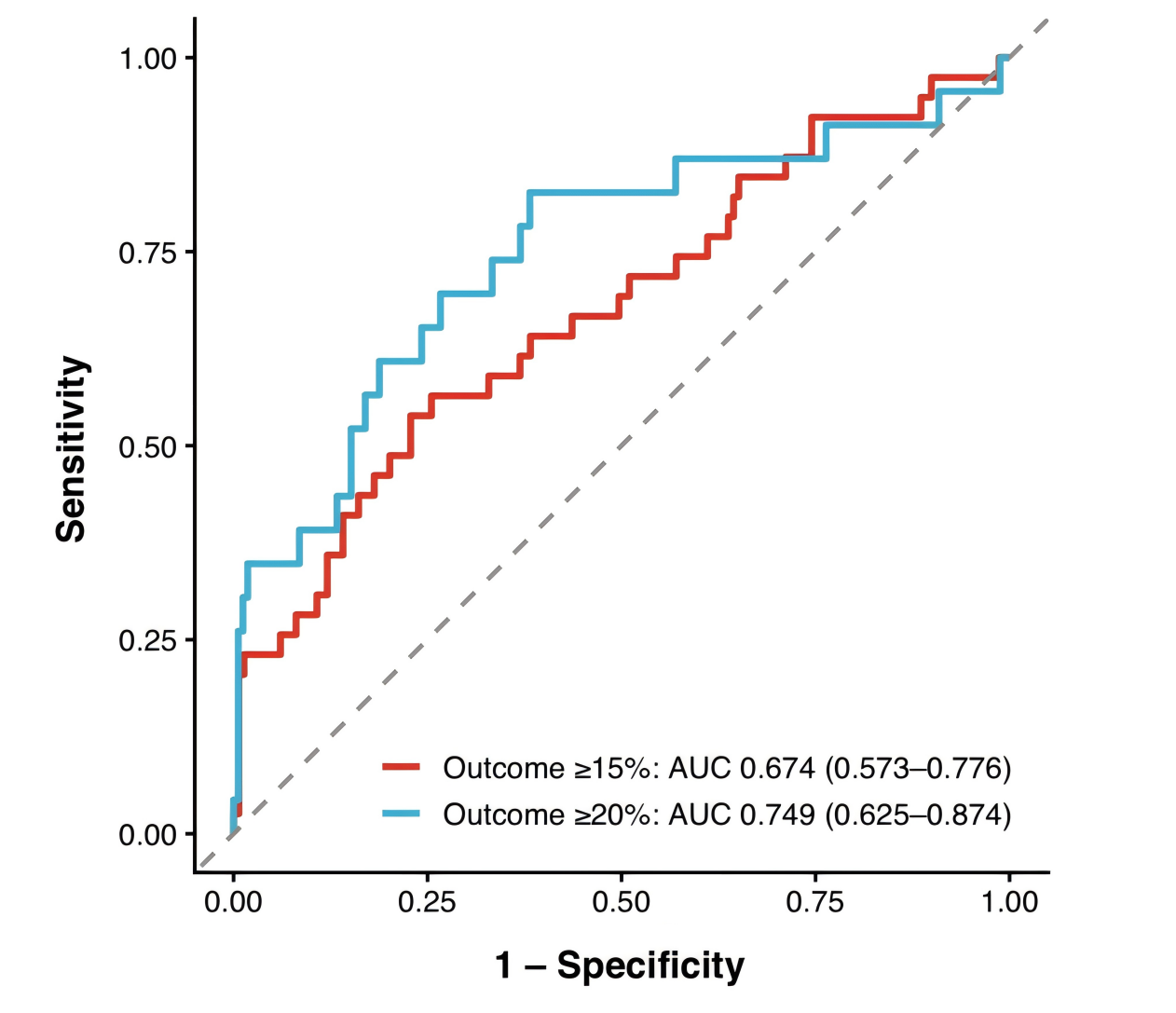


**Supplementary Figure 3.** ROC curves of the multivariate models refitted for stricter definitions of renal recovery.

AUC, area under the curve; CI, confidence interval.


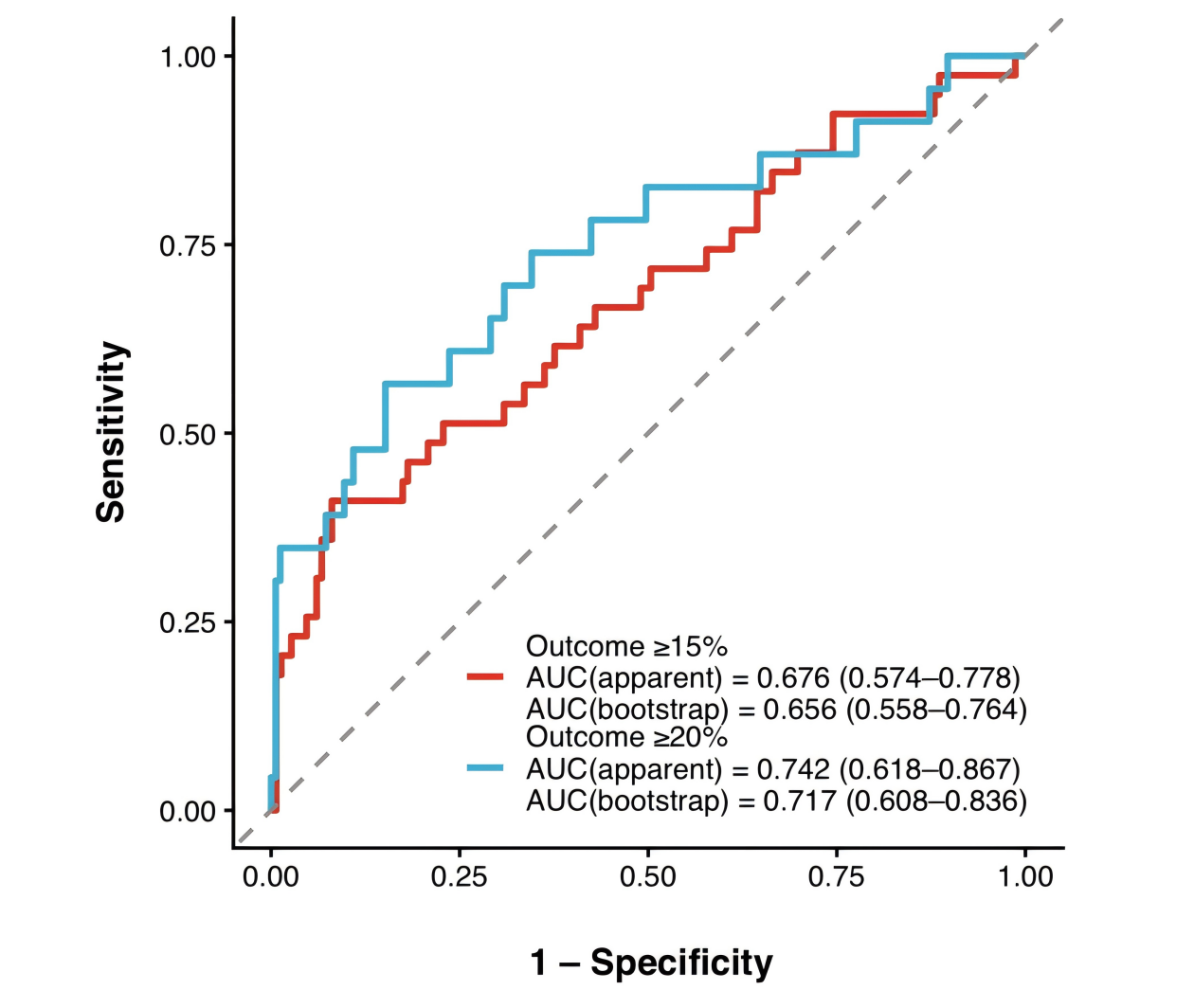

Supplement: Supplementary file 1 — Table S1: Sensitivity analysis of the multivariate logistic regression model for predicting renal function improvement using stricter thresholds. Table S2: Stratified multivariate logistic regression analysis of predictors for renal function improvement based on baseline eGFR levels. Figure S1: Correlation matrix of baseline renal function markers. Figure S2: Validation of the fixed multivariate model (derived from the > 10% improvement threshold) for predicting stricter renal recovery outcomes. Figure S3: ROC curves of the multivariate models refitted for stricter definitions of renal recovery. [file KJM2-9999-e70268-s001.docx]
